# The Role of a 1064-nm Picosecond Laser in Treating Prolonged Bruising Secondary to Liquid Polycaprolactone Injection

**DOI:** 10.7759/cureus.117667

**Published:** 2026-10-09

**Authors:** Zenus Saleh

**Affiliations:** 1 Dermatology, Soliman Fakeeh Hospital, Riyadh, SAU

**Keywords:** filler injection, laser therapy, nd:yag laser, pico laser, poly(ε-caprolactone)

## Abstract

Liquid-form polycaprolactone (PCL) is an increasingly utilized dermal biostimulator in aesthetic dermatology. We report the case of a 72-year-old man who developed persistent infraorbital bruising following bilateral tear trough injection of liquid PCL. Owing to bruising persisting over six weeks, the patient underwent four weekly sessions of long-pulsed 1064-nm Nd laser therapy with minimal improvement. Subsequently, treatment with a 1064-nm picosecond laser was initiated, followed by resolution of the bruising after two sessions. To our knowledge, this represents the eighth reported case of prolonged bruising after liquid PCL injection and the sixth involving the tear trough region. We hypothesize that sequestration of hemoglobin degradation products within the PCL scaffold may contribute to prolonged bruising. Picosecond laser therapy was associated with clinical improvement in this patient and may represent a potentially useful therapeutic approach for this uncommon complication; however, its efficacy, optimal parameters, mechanism of action, and safety require confirmation in additional patients and controlled studies.

## Introduction

Polycaprolactone (PCL) is a novel dermal biostimulator in aesthetic medicine. In its liquid formulation, PCL is homogeneously distributed within the dermis, allowing for even tissue integration and uniform collagen induction [1]. This property of liquid-form PCL has led lately to an increase in its use in many countries around the world. Although regarded as generally safe, long-term bruising has been reported previously as one possible side effect of its use [2]. Herein, we present a new case of prolonged bruising following liquid PCL injection in the tear troughs that improved following treatment with a 1064-nm picosecond laser.

## Case presentation

A 72-year-old man with Fitzpatrick skin phototype III and a history of hypertension, who was not receiving anticoagulant or antiplatelet therapy, presented for treatment of lower eyelid laxity. The patient had no previous injectable, energy-based, or other aesthetic treatment in the infraorbital region prior to presentation. In December 2025, he underwent bilateral infraorbital injection of liquid-form PCL (GOURI®, Dexlevo, South Korea), administered as 0.5 mL per side in undiluted form. After skin antisepsis with 10% povidone-iodine and application of topical anesthetic cream (lidocaine 4% cream) for 30 minutes, injections were performed with a 29-gauge, 1/2-inch needle inserted perpendicular to the skin at two points per side, located just inferior to the infraorbital rim and evenly spaced from the medial canthal line to the mid-pupillary line. The product was deposited in the supraperiosteal plane. Aspiration was performed for 10 seconds before each injection and was negative. The product was injected as static depots with the needle stationary during needle withdrawal, at approximately 0.25 mL per depot, delivered slowly over approximately 10 seconds. No blanching, livedo, or disproportionate pain was observed during or after injection. An immediate post-procedural ecchymosis developed in the left infraorbital region, presenting as a discrete purple-blue bruise. Initial management consisted of application of pressure and cold compresses at the site of the bruise. The patient was prescribed topical arnica cream for home application twice daily for two weeks. Despite this conservative approach, the bruising remained clinically evident at follow-up in January 2026, approximately six weeks after the index procedure (Figure 1A). Given the patient's age and the blue-purple coloration of the lesion, treatment was initiated with a long-pulsed neodymium-doped yttrium aluminum garnet (Nd) 1064-nm laser (Cutera Excel V, Cutera Inc., Brisbane, CA, USA), using a fluence of 70 J/cm², a 6-mm spot size, a pulse duration of 40 ms, and three passes per session with non-overlapping pulses and a five-second interval between passes. The device's integrated contact-cooled sapphire tip was used, along with pretreatment topical anesthesia with lidocaine 4% cream for 30 minutes. The treatment endpoint was mild erythema. The patient completed four weekly sessions under this protocol (January and February 2026). The patient was then lost to follow-up until March 2026, when the bruising was still present (Figure 1B). Given this limited response, treatment was switched to a 1064-nm picosecond laser therapy. Two sessions, spaced one week apart, were performed in March 2026 (Figures 1B, 1C), using a picosecond laser platform (Candela PicoWay laser, Candela Corporation, Marlborough, MA, USA) fitted with the Zoom handpiece. The treatment parameters were a pulse duration of 450 ps, a fluence of 0.6 J/cm², an 8-mm spot size, a frequency of 5 Hz, and four passes per session. No topical anesthesia was required, and an ice pack was used for cooling after treatment. The clinical endpoint was mild erythema. The response to this modality was markedly more favorable than that achieved with the long-pulsed Nd laser. One week after the second session, complete resolution of the infraorbital discoloration was observed on clinical examination (Figure 1D), with no reported adverse effects. During all laser sessions, ocular protection was ensured with intraocular stainless-steel corneal shields placed after topical anesthetic eye drops, and all personnel wore protective eyewear rated for 1064-nm wavelengths.

**Figure 1:**
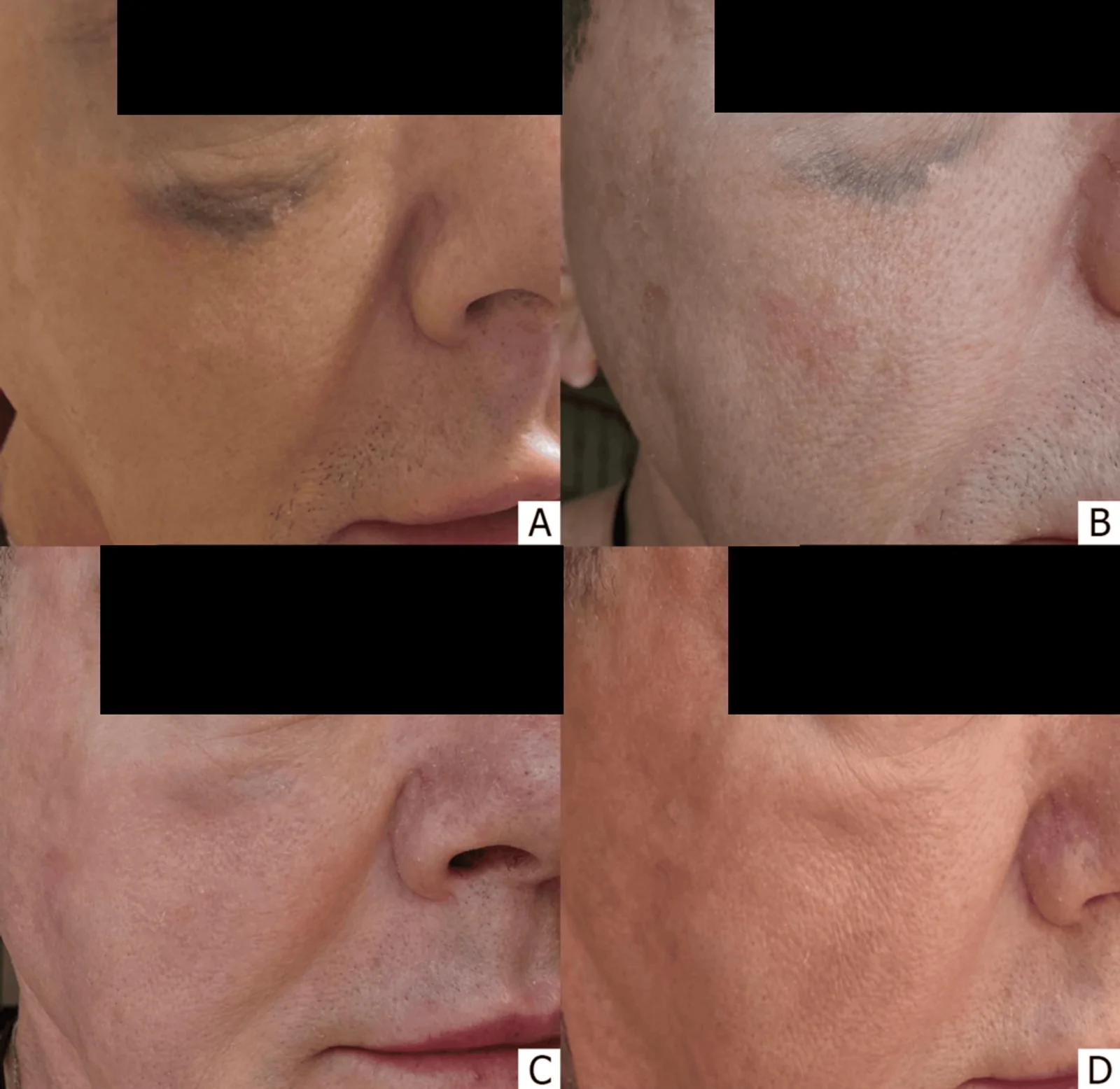
Polycaprolactone (PCL)-induced bruising (A) PCL-induced bruising in January 2026. (B) Persistent bruising in March 2026. (C) Marked improvement after one pico laser session (March 2026). (D) Resolution of the bruising one week after the second pico session (March 2026).

## Discussion

Bruising results from vascular injury leading to extravasation of red blood cells into the surrounding tissues, followed by progressive hemoglobin degradation into biliverdin and bilirubin, accounting for the characteristic color changes over time [3]. Bruising is among the most common adverse effects following periorbital filler injection. Reported incidence rates range from approximately 89% to 100% across studies, depending on the injection technique and patient-related factors [2]. These events are typically mild and self-limited, resolving within several days to two weeks [4].

Prolonged bruising (discoloration persisting beyond two weeks) after liquid-form PCL injection appears to represent a rare but clinically relevant entity. To date, only one case series has reported long-term bruising associated with liquid PCL, describing seven cases, five of which involved the infraorbital region [5]. To our knowledge, the present case represents the eighth documented case of prolonged bruising after liquid PCL injection and the sixth affecting the tear trough region. In that series, persistent bruising was hypothesized to result from sequestration of hemoglobin degradation products within the dense PCL scaffold, as well as potential contributions from the Tyndall effect in thin-skinned regions such as the infraorbital area. The unilateral occurrence of bruising despite an identical technique on both sides suggests an incidental needle injury to a small vessel rather than a technique-related cause.

Different lasers and light-based therapies have previously been shown to be effective in accelerating the resolution of postprocedural bruising, reducing healing time from approximately 14 days to 24-48 hours [4]. The pulsed dye laser (PDL; 585/595 nm), which targets oxyhemoglobin as its chromophore, remains the most extensively studied modality and is primarily effective for early, erythematous bruising (24-48 hours post-procedure) [4]. The long-pulsed Nd 1064-nm laser, whose wavelength is preferentially absorbed by deoxyhemoglobin, provides deeper dermal penetration with lower epidermal melanin absorption, making it particularly suitable for deeper or more persistent bruising and for use across a broader range of skin phototypes [3,6]. Narrowband IPL (500-600 nm), by targeting bilirubin and biliverdin-hemoglobin degradation derivatives rather than only oxyhemoglobin, demonstrates efficacy across both early and later stages of bruising [3]. Picosecond lasers, which predominantly exert photomechanical rather than photothermal effects, enable efficient pigment disruption with minimal thermal injury. A phase-adapted protocol described by Yi et al. demonstrates that picosecond laser therapy is effective across the full temporal spectrum of ecchymosis [6].

In this context, the long-pulsed Nd laser sessions employed in our case were intended to promote resolution of the purple-blue bruising. However, complete resolution was not achieved, as the patient demonstrated modest improvement after four treatment sessions. Notably, a clinically significant response was observed only following the initiation of picosecond laser therapy. This differential response is consistent with, but does not confirm, the hypothesis proposed by Byeon et al., namely, the entrapment of pigment degradation products within the PCL scaffold. Within the scaffold, hemoglobin degradation products may remain sequestered and be less readily accessible to macrophages, which may explain the prolonged bruising associated with liquid PCL injections. Byeon et al. proposed that lipophilic free heme and its catabolites, such as biliverdin and bilirubin, may become sequestered within the hydrophobic domains of the PCL scaffold [5]. However, bilirubin and biliverdin absorb predominantly in the visible spectrum and are therefore unlikely to have been the principal targets at 1064 nm. We hypothesize that two chromophores were relevant in our patient. The first is residual deoxyhemoglobin, the main target of the photothermal long-pulsed Nd laser, which may explain the partial initial response. The second is hemosiderin, the insoluble, particulate, iron-containing product of hemoglobin degradation, whose clearance depends on macrophage phagocytosis. Particulate targets of this kind are well suited to the photomechanical effect of picosecond pulses; Q-switched and picosecond 1064-nm lasers have been used successfully to clear iron-related cutaneous pigmentation [7-9], and Byeon et al. and Yi et al. likewise attributed the effects of picosecond lasers to photomechanical disruption of hemosiderin [5,6]. We therefore hypothesize that the picosecond laser fragmented sequestered hemosiderin into smaller particles that were more amenable to macrophage and lymphatic clearance. Deep dermal hemosiderin viewed through thin infraorbital skin may also appear bluish rather than brown. Whether the PCL scaffold itself was disrupted remains speculative. PCL is not a recognized chromophore at 1064 nm, Byeon et al. classified picosecond lasers as pigment-fragmentation rather than scaffold-disrupting modalities [5], and, to our knowledge, no experimental or clinical data have demonstrated disruption of liquid PCL by a picosecond laser at the low fluence used here (0.6 J/cm², 8-mm spot). Any effect on the scaffold would therefore have been indirect. In the series by Byeon et al., treatments were grouped into four mechanistic strategies: pigment fragmentation (picosecond, Q-switched, and long-pulsed Nd lasers, IPL, and PDL), thermal loosening of the scaffold (radiofrequency, HIFU), mechanical dispersion, and enzymatic degradation. Picosecond laser therapy alone was used effectively in two of seven cases, predominantly at 532 nm; the 1064-nm wavelength was used in only one of these cases, at a higher fluence (2.6 J/cm², 5-mm spot) than in our patient [5]. Similarly, in our case, picosecond laser therapy was associated with clinical improvement in prolonged bruising after liquid PCL, without adverse effects.

This report has limitations. Neither dermoscopic nor histopathologic evaluation was performed; therefore, the exact nature of the persistent discoloration at the time of picosecond treatment could not be objectively confirmed. Based on its evolution from an immediate post-injection ecchymosis, its strictly unilateral distribution despite symmetric injection technique, and its partial response to long-pulsed Nd, we consider retained hemoglobin and its degradation products within the PCL scaffold to be the most likely explanation, although an optical contribution from the Tyndall effect cannot be excluded. Most importantly, as this is a single uncontrolled case, the temporal association between picosecond treatment and resolution does not establish causality. Prolonged bruising after liquid PCL injection may persist for several months and has been observed to resolve in parallel with scaffold degradation [5]. Since the bruising in our patient had been present for approximately 13 weeks by the time of the second picosecond session, spontaneous clearance coinciding with treatment is a plausible alternative explanation. A delayed effect of the preceding long-pulsed Nd sessions likewise cannot be excluded. The patient was lost to follow-up during the 24-day interval between the last Nd session (February) and the first picosecond session (March), so the course of the bruising during this period was not documented. However, the bruising appeared clinically unchanged in March compared with its appearance at the end of the Nd treatment, whereas resolution was observed seven days after the second picosecond session. Controlled studies are needed to determine whether picosecond laser therapy truly accelerates the resolution of prolonged bruising after liquid PCL injection.

## Conclusions

In conclusion, physicians should be aware that, unlike conventional bruising, PCL-associated prolonged bruising may be less dependent on simple vascular injury and more related to impaired clearance of hemoglobin degradation products within a polymer scaffold. This uncommon complication may follow a prolonged course and respond poorly to standard conservative measures. In our patient, the resolution observed after picosecond laser treatment suggests a possible role of this laser in managing prolonged bruising induced by liquid PCL. However, its efficacy, optimal parameters, mechanism of action, and safety require confirmation in additional patients and controlled studies.
